# The genome sequence of a bluebottle fly, *Calliphora vicina *(Linnaeus, 1758)

**DOI:** 10.12688/wellcomeopenres.22469.1

**Published:** 2024-06-24

**Authors:** Olga Sivell, Natural History Museum Genome Acquisition Lab, Darwin Tree of Life Barcoding collective, Wellcome Sanger Institute Tree of Life Management, Samples and Laboratory team, Wellcome Sanger Institute Scientific Operations: Sequencing Operations, Wellcome Sanger Institute Tree of Life Core Informatics team, Tree of Life Core Informatics collective, Darwin Tree of Life Consortium

**Affiliations:** 1 Natural History Museum, London, England, UK

**Keywords:** Calliphora vicina, bluebottle blow fly, genome sequence, chromosomal, Diptera

## Abstract

We present a genome assembly from an individual female *Calliphora vicina* (bluebottle blow fly; Arthropoda; Insecta; Diptera; Calliphoridae). The genome sequence is 706.5 megabases in span. Most of the assembly is scaffolded into 6 chromosomal pseudomolecules, including the X sex chromosome. The mitochondrial genome has also been assembled and is 16.72 kilobases in length. Gene annotation of this assembly on Ensembl identified 13,436 protein coding genes.

## Species taxonomy

Eukaryota; Opisthokonta; Metazoa; Eumetazoa; Bilateria; Protostomia; Ecdysozoa; Panarthropoda; Arthropoda; Mandibulata; Pancrustacea; Hexapoda; Insecta; Dicondylia; Pterygota; Neoptera; Endopterygota; Diptera; Brachycera; Muscomorpha; Eremoneura; Cyclorrhapha; Schizophora; Calyptratae; Oestroidea; Calliphoridae; Calliphorinae; *Calliphora*; *Calliphora vicina* (Linnaeus, 1758) (NCBI:txid7373).

## Background

*Calliphora vicina* (Linnaeus, 1758) is a metallic blue fly from the family Calliphoridae (blow flies). This species can be identified by the following characters: the anterior thoracic spiracle is orange, the gena is orange and covered in black hairs, and is clearly demarcated from the black postgena, the lower calypter is dark with a white rim and has dark hairs on its upper surface, the basicosta is normally pale (cream, yellow or orange) but occasionally can be dark, though it is never entirely black ( Rognes, 1991; Sivell, 2021).

This species is widely distributed and occurs worldwide, except in Greenland ( Draber-Mońko, 2004; Rognes, 1991). It is the most common and widely distributed of British bluebottles ( Davies, 1990; Davies, 1999; Davies & Laurence, 1992; MacLeod, 1943; MacLeod, 1963; MacLeod & Donnelly, 1956; Sivell, 2021). Adults can be encountered throughout the year, on sunny winter days resting on fences and visiting carrion and faeces. The species is highly synanthropic and commonly found in houses ( Rognes, 1991; Sivell, 2021).

The adults are attracted to carrion, faeces, stinkhorn fungus *Phallus impudicus* Linnaeus, 1753, flowering plants and ripe fruit; they feed mainly on sugar and are important pollinators. The females require a protein meal to reach maturity and produce eggs ( Rivers & Dahlem, 2014). Oviposition takes place on carrion, with a preference for areas away from direct sunlight to avoid desiccation of the eggs and larvae (e.g. natural body openings). The fly can also oviposit on a live host, particularly if wounded or soiled. The larvae can feed on live tissues in a condition called myiasis. This may result in the host’s death. *Calliphora vicina* was reported from variety of vertebrate hosts including humans, but it only plays minor role in sheep strike ( Haddow & Thomson, 1937; MacLeod, 1943; Morris & Titchener, 1997; Taylor *et al.*, 2013). This species is of great forensic importance and its developmental rates have been researched using populations from North America ( Anderson, 2000; Greenberg, 1991; Hans & Vanlaerhoven, 2021; Kamal, 1958; Niederegger *et al.*, 2010; Niederegger *et al.*, 2013) and Europe ( Marchenko, 2001; Niederegger *et al.*, 2010; Niederegger *et al.*, 2013) including from Britain ( Davies & Ratcliffe, 1994; Donovan *et al.*, 2006). This species is also a vector of mechanically transmitted diseases ( Fischer *et al.*, 2001; Fischer *et al.*, 2004; Maldonado & Centeno, 2003).

The genome sequence we present here is based on one female specimen from Wigmore Park, Luton (51.88, –0.36), collected by OS on 17/06/2020. The specimen (NHMUK014111059) was identified using Sivell (2021). The chromosomally complete genome sequence for *Calliphora vicina* has been generated as part of the Darwin Tree of Life project, a collaborative effort to sequence all named eukaryotic species in the Atlantic Archipelago of Britain and Ireland. It will aid research on the phylogeny, taxonomy, biology, and ecology of the species.

## Genome sequence report

**Figure 1.**
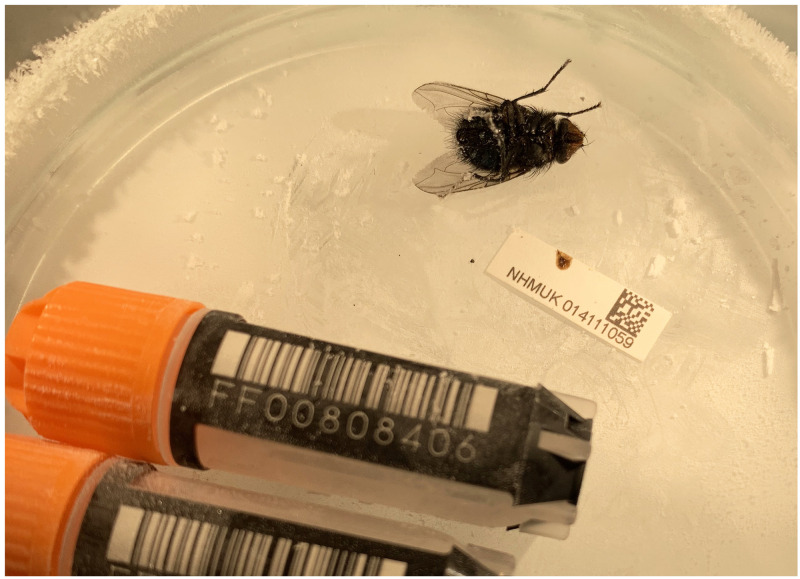
Photograph of the *Calliphora vicina* (NHMUK014111059; idCalVici1) specimen used for genome sequencing.

The genome was sequenced from a female *Calliphora vicina* ( Figure 1) collected from Luton, England, UK (51.88, –0.37). A total of 50-fold coverage in Pacific Biosciences single-molecule HiFi long reads was generated. Primary assembly contigs were scaffolded with chromosome conformation Hi-C data. Manual assembly curation corrected 73 missing joins or mis-joins and removed 12 haplotypic duplications, reducing the assembly length by 3.88% and the scaffold number by 20.27%, and increasing the scaffold N50 by 7.00%.

**Figure 2.**
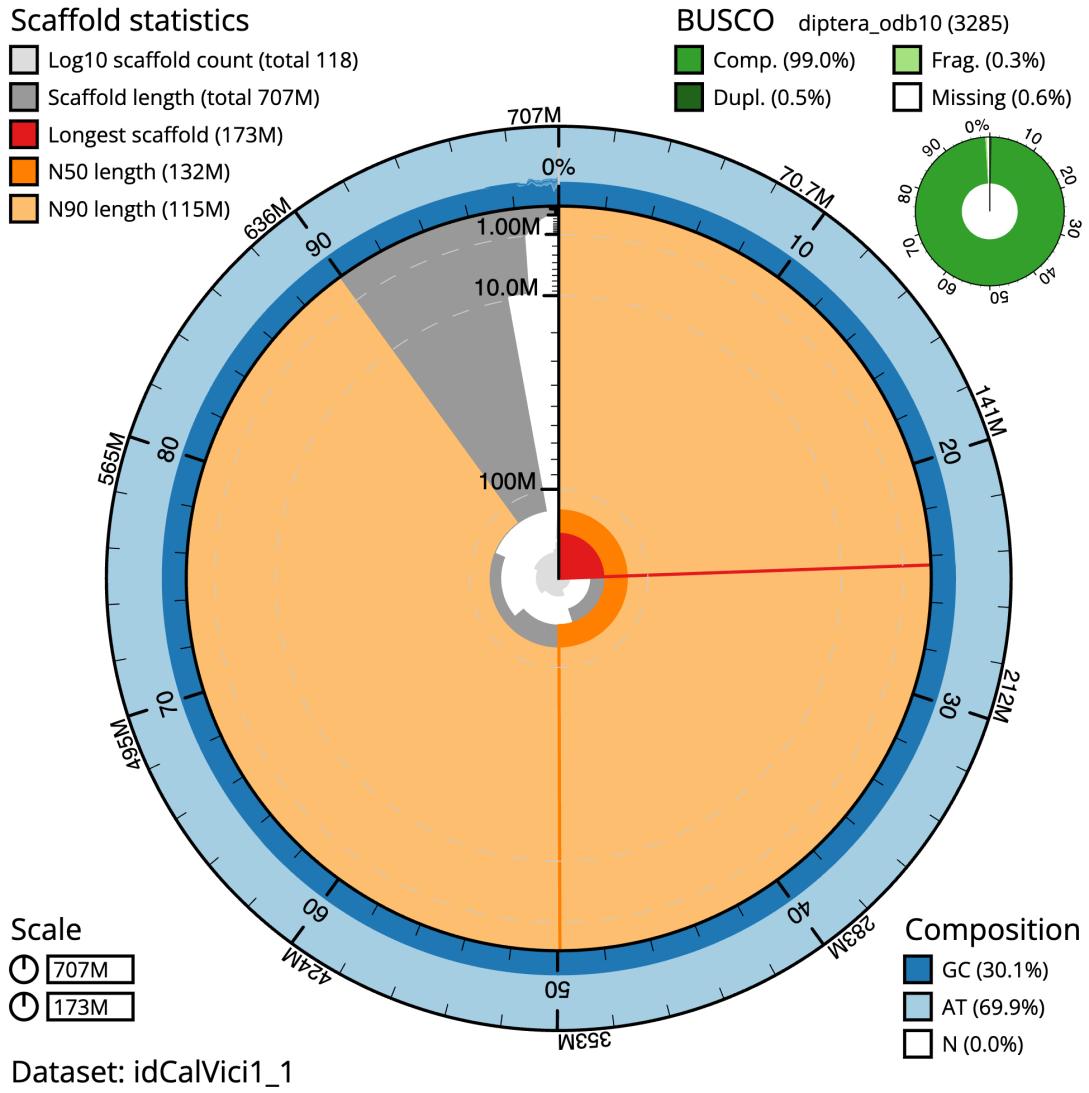
Genome assembly of *Calliphora vicina*, idCalVici1.1: metrics. The BlobToolKit snail plot shows N50 metrics and BUSCO gene completeness. The main plot is divided into 1,000 size-ordered bins around the circumference with each bin representing 0.1% of the 706,541,155 bp assembly. The distribution of sequence lengths is shown in dark grey with the plot radius scaled to the longest sequence present in the assembly (172,799,725 bp, shown in red). Orange and pale-orange arcs show the N50 and N90 sequence lengths (131,702,511 and 114,622,126 bp), respectively. The pale grey spiral shows the cumulative sequence count on a log scale with white scale lines showing successive orders of magnitude. The blue and pale-blue area around the outside of the plot shows the distribution of GC, AT and N percentages in the same bins as the inner plot. A summary of complete, fragmented, duplicated and missing BUSCO genes in the diptera_odb10 set is shown in the top right. An interactive version of this figure is available at https://blobtoolkit.genomehubs.org/view/Calliphora%20vicina/dataset/idCalVici1_1/snail.

**Figure 3.**
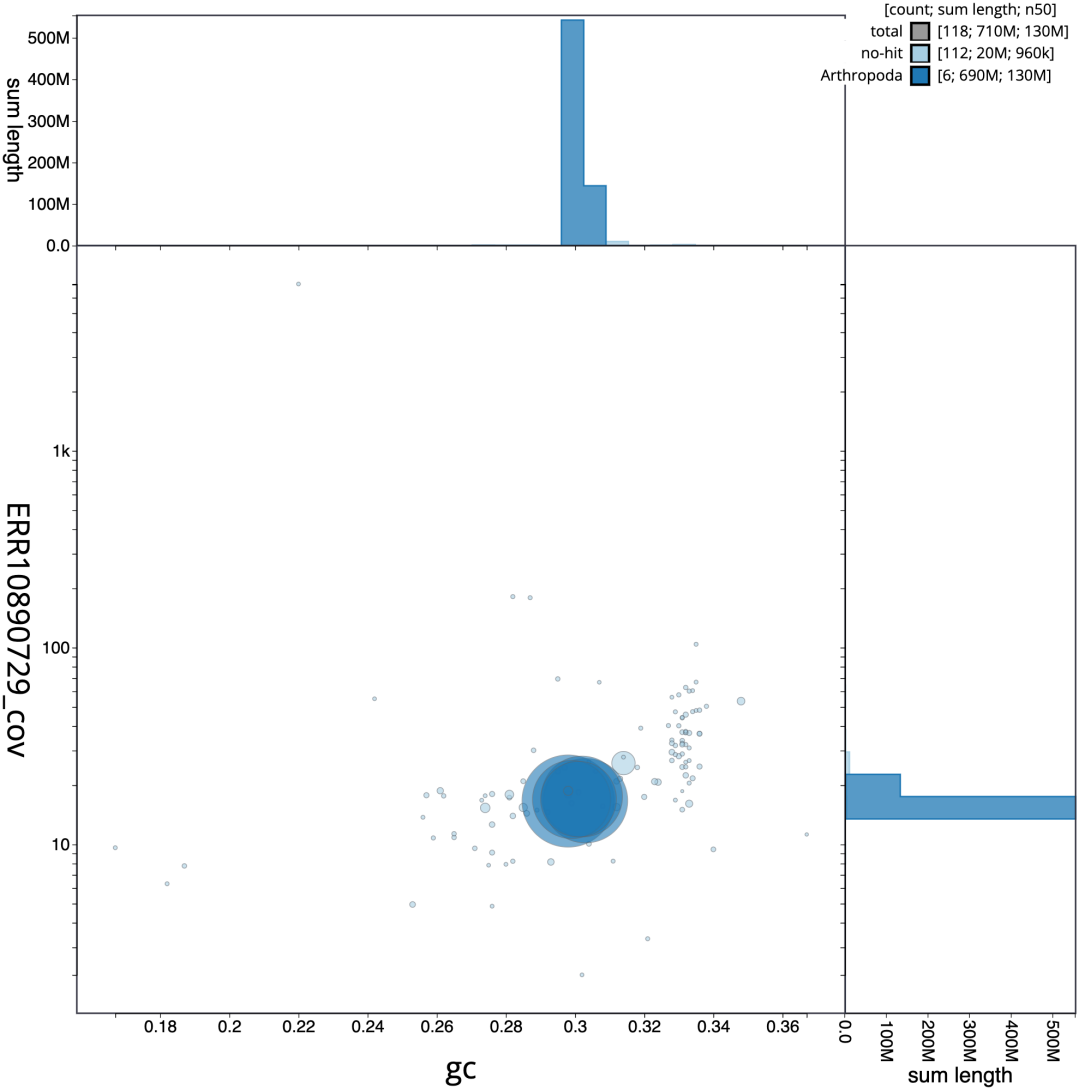
Genome assembly of *Calliphora vicina*, idCalVici1.1: BlobToolKit GC-coverage plot. Sequences are coloured by phylum. Circles are sized in proportion to sequence length. Histograms show the distribution of sequence length sum along each axis. An interactive version of this figure is available at https://blobtoolkit.genomehubs.org/view/Calliphora vicina/dataset/idCalVici1_1/blob.

**Figure 4.**
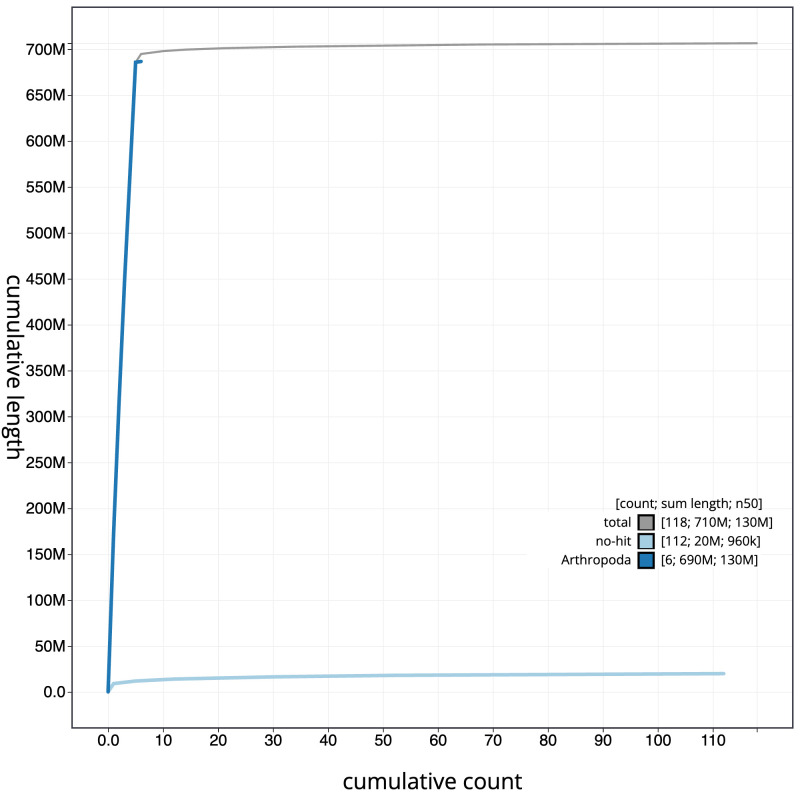
Genome assembly of *Calliphora vicina*, idCalVici1.1: BlobToolKit cumulative sequence plot. The grey line shows cumulative length for all sequences. Coloured lines show cumulative lengths of sequences assigned to each phylum using the buscogenes taxrule in the BlobToolKit environment. An interactive version of this figure is available at https://blobtoolkit.genomehubs.org/view/Calliphora%20vicina/dataset/idCalVici1_1/cumulative.

**Figure 5.**
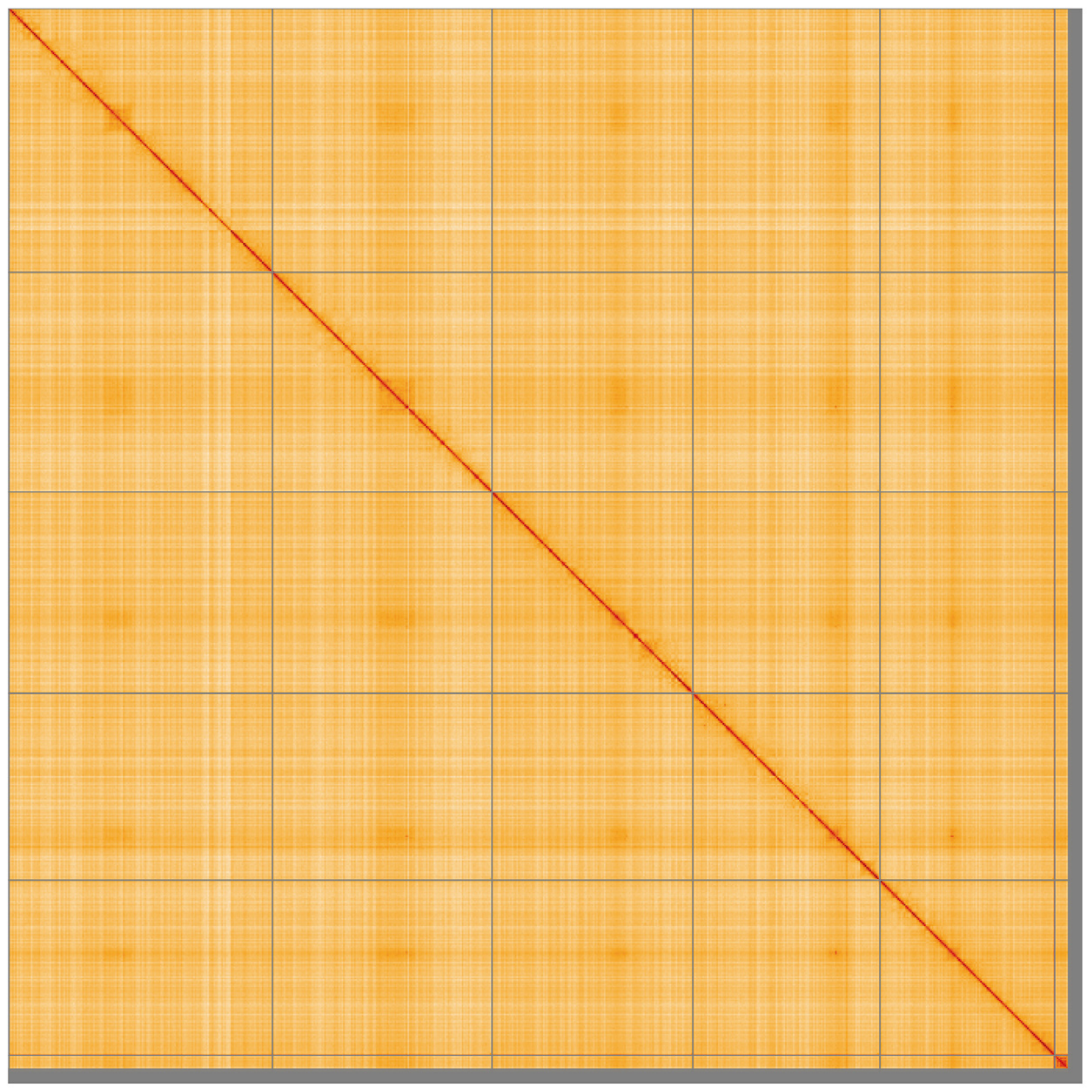
Genome assembly of *Calliphora vicina*, idCalVici1.1: Hi-C contact map of the idCalVici1.1 assembly, visualised using HiGlass. Chromosomes are shown in order of size from left to right and top to bottom. An interactive version of this figure may be viewed at https://genome-note-higlass.tol.sanger.ac.uk/l/?d=JYSjdNSRRIC0XJ3z8-tzbg.

The final assembly has a total length of 706.5 Mb in 117 sequence scaffolds with a scaffold N50 of 131.7 Mb ( Table 1). The snail plot in Figure 2 provides a summary of the assembly statistics, while the distribution of assembly scaffolds on GC proportion and coverage is shown in Figure 3. The cumulative assembly plot in Figure 4 shows curves for subsets of scaffolds assigned to different phyla. Most (98.34%) of the assembly sequence was assigned to 6 chromosomal-level scaffolds, representing 5 autosomes and the X sex chromosome. Chromosome-scale scaffolds confirmed by the Hi-C data are named in order of size ( Figure 5; Table 2). Chromosome X was assigned based on synteny to *Calliphora vomitoria* (GCA_942486065.2) ( Sivell *et al.*, 2023). While not fully phased, the assembly deposited is of one haplotype. Contigs corresponding to the second haplotype have also been deposited. The mitochondrial genome was also assembled and can be found as a contig within the multifasta file of the genome submission.

**Table 1.** Genome data for *Calliphora vicina*, idCalVici1.1.

Project accession data		
Assembly identifier	idCalVici1.1	
Species	*Calliphora vicina*	
Specimen	idCalVici1	
NCBI taxonomy ID	7373	
BioProject	PRJEB59784	
BioSample ID	SAMEA7521395	
Isolate information	idCalVici1, female: head and thorax (DNA and Hi-C sequencing); abdomen (RNA sequencing)	
Assembly metrics *		*Benchmark*
Consensus quality (QV)	60.6	*≥ 50*
*k*-mer completeness	100.0%	*≥ 95%*
BUSCO **	C:99.0%[S:98.5%,D:0.5%], F:0.3%,M:0.7%,n:3,285	*C ≥ 95%*
Percentage of assembly mapped to chromosomes	98.34%	*≥ 95%*
Sex chromosomes	X	*localised homologous pairs*
Organelles	Mitochondrial genome: 16.72 kb	*complete single alleles*
Raw data accessions		
PacificBiosciences Sequel IIe	ERR10879929, ERR10880460	
Hi-C Illumina	ERR10890731	
PolyA RNA-Seq Illumina	ERR11641127	
Genome assembly		
Assembly accession	GCA_958450345.1	
*Accession of alternate haplotype*	GCA_958301635.1	
Span (Mb)	706.5	
Number of contigs	251	
Contig N50 length (Mb)	35.3	
Number of scaffolds	117	
Scaffold N50 length (Mb)	131.7	
Longest scaffold (Mb)	172.8	
Genome annotation		
Number of protein-coding genes	13,436	
Number of non-coding genes	4,947	
Number of gene transcripts	26,159	

* Assembly metric benchmarks are adapted from column VGP-2020 of “Table 1: Proposed standards and metrics for defining genome assembly quality” from Rhie *et al.* (2021). ** BUSCO scores based on the diptera_odb10 BUSCO set using version v5.3.2. C = complete [S = single copy, D = duplicated], F = fragmented, M = missing, n = number of orthologues in comparison. A full set of BUSCO scores is available at https://blobtoolkit.genomehubs.org/view/Calliphora vicina/dataset/idCalVici1_1/busco.

**Table 2.** Chromosomal pseudomolecules in the genome assembly of *Calliphora vicina*, idCalVici1.

INSDC accession	Chromosome	Length (Mb)	GC%
OY288232.1	1	172.8	30.0
OY288233.1	2	143.96	30.5
OY288234.1	3	131.7	30.0
OY288235.1	4	122.7	30.0
OY288236.1	5	114.62	30.0
OY288237.1	X	9.04	31.5
OY288238.1	MT	0.02	22.0

The estimated Quality Value (QV) of the final assembly is 60.6 with *k*-mer completeness of 100.0%, and the assembly has a BUSCO v5.3.2 completeness of 99.0% (single = 98.5%, duplicated = 0.5%), using the diptera_odb10 reference set ( *n* = 3,285).

Metadata for specimens, BOLD barcode results, spectra estimates, sequencing runs, contaminants and pre-curation assembly statistics are given at https://links.tol.sanger.ac.uk/species/7373.

## Genome annotation report

The *Calliphora vicina* genome assembly (GCA_958450345.1) was annotated at the European Bioinformatics Institute (EBI) on Ensembl Rapid Release. The resulting annotation includes 26,159 transcribed mRNAs from 13,436 protein-coding and 4,947 non-coding genes ( Table 1; https://rapid.ensembl.org/Calliphora_vicina_GCA_958450345.1/Info/Index).

## Methods

### Sample acquisition and nucleic acid extraction

A female *Calliphora vicina* (specimen ID NHMUK014111059, ToLID idCalVici1) was collected from Wigmore Park, Luton, England, UK (latitude 51.88, longitude –0.37) on 2020-06-17 by netting. The specimen was collected and identified by Olga Sivell (Natural History Museum, London) and preserved on dry ice.

The workflow for high molecular weight (HMW) DNA extraction at the Wellcome Sanger Institute (WSI) Tree of Life Core Laboratory includes a sequence of core procedures: sample preparation; sample homogenisation, DNA extraction, fragmentation, and clean-up. In sample preparation, the idCalVici1 sample was weighed and dissected on dry ice ( Jay *et al.*, 2023). Tissue from the head and thorax was homogenised using a PowerMasher II tissue disruptor ( Denton *et al.*, 2023a). HMW DNA was extracted using the Manual MagAttract v1 protocol ( Strickland *et al.*, 2023b). DNA was sheared into an average fragment size of 12–20 kb in a Megaruptor 3 system with speed setting 30 ( Todorovic *et al.*, 2023). Sheared DNA was purified by solid-phase reversible immobilisation ( Strickland *et al.*, 2023a): in brief, the method employs a 1.8X ratio of AMPure PB beads to sample to eliminate shorter fragments and concentrate the DNA. The concentration of the sheared and purified DNA was assessed using a Nanodrop spectrophotometer and Qubit Fluorometer and Qubit dsDNA High Sensitivity Assay kit. Fragment size distribution was evaluated by running the sample on the FemtoPulse system.

RNA was extracted from abdomen tissue of idCalVici1 in the Tree of Life Laboratory at the WSI using the RNA Extraction: Automated MagMax™ *mir*Vana protocol ( do Amaral *et al.*, 2023). The RNA concentration was assessed using a Nanodrop spectrophotometer and a Qubit Fluorometer using the Qubit RNA Broad-Range Assay kit. Analysis of the integrity of the RNA was done using the Agilent RNA 6000 Pico Kit and Eukaryotic Total RNA assay.

Protocols developed by the WSI Tree of Life laboratory are publicly available on protocols.io ( Denton *et al.*, 2023b).

### Sequencing

Pacific Biosciences HiFi circular consensus DNA sequencing libraries were constructed according to the manufacturers’ instructions. Poly(A) RNA-Seq libraries were constructed using the NEB Ultra II RNA Library Prep kit. DNA and RNA sequencing was performed by the Scientific Operations core at the WSI on Pacific Biosciences Sequel II (HiFi) and Illumina NovaSeq 6000 (RNA-Seq) instruments. Hi-C data were also generated from head and thorax tissue of idCalVici1 using the Arima2 kit and sequenced on the Illumina NovaSeq 6000 instrument.

### Genome assembly and curation

Assembly was carried out with Hifiasm ( Cheng *et al.*, 2021) and haplotypic duplication was identified and removed with purge_dups ( Guan *et al.*, 2020). The assembly was then scaffolded with Hi-C data ( Rao *et al.*, 2014) using YaHS ( Zhou *et al.*, 2023). The assembly was checked for contamination and corrected using the TreeVal pipeline ( Pointon *et al.*, 2023). Manual curation was performed using JBrowse2 ( Diesh *et al.*, 2023), HiGlass ( Kerpedjiev *et al.*, 2018) and PretextView ( Harry, 2022). The mitochondrial genome was assembled using MitoHiFi ( Uliano-Silva *et al.*, 2023), which runs MitoFinder ( Allio *et al.*, 2020) or MITOS ( Bernt *et al.*, 2013) and uses these annotations to select the final mitochondrial contig and to ensure the general quality of the sequence.

### Evaluation of final assembly

A Hi-C map for the final assembly was produced using bwa-mem2 ( Vasimuddin *et al.*, 2019) in the Cooler file format ( Abdennur & Mirny, 2020). To assess the assembly metrics, the *k*-mer completeness and QVy consensus quality values were calculated in Merqury ( Rhie *et al.*, 2020). This work was done using Nextflow ( Di Tommaso *et al.*, 2017) DSL2 pipelines “sanger-tol/readmapping” ( Surana *et al.*, 2023a) and “sanger-tol/genomenote” ( Surana *et al.*, 2023b). The genome was analysed within the BlobToolKit environment ( Challis *et al.*, 2020) and BUSCO scores ( Manni *et al.*, 2021; Simão *et al.*, 2015) were calculated.

Table 3 contains a list of relevant software tool versions and sources.

**Table 3.** Software tools: versions and sources.

Software tool	Version	Source
BlobToolKit	4.2.1	https://github.com/blobtoolkit/blobtoolkit
BUSCO	5.3.2	https://gitlab.com/ezlab/busco
Hifiasm	0.16.1-r375	https://github.com/chhylp123/hifiasm
HiGlass	1.11.6	https://github.com/higlass/higlass
Merqury	MerquryFK	https://github.com/thegenemyers/MERQURY.FK
MitoHiFi	2	https://github.com/marcelauliano/MitoHiFi
PretextView	0.2	https://github.com/wtsi-hpag/PretextView
purge_dups	1.2.3	https://github.com/dfguan/purge_dups
sanger-tol/genomenote	v1.0	https://github.com/sanger-tol/genomenote
sanger-tol/readmapping	1.1.0	https://github.com/sanger-tol/readmapping/tree/1.1.0
TreeVal	1.0.0	https://github.com/sanger-tol/treeval
YaHS	yahs-1.1.91eebc2	https://github.com/c-zhou/yahs

### Genome annotation

The Ensembl Genebuild annotation system ( Aken *et al.*, 2016) was used to generate annotation for the *Calliphora vicina* assembly (GCA_958450345.1) in Ensembl Rapid Release at the EBI. Annotation was created primarily through alignment of transcriptomic data to the genome, with gap filling via protein-to-genome alignments of a select set of proteins from UniProt ( Bateman *et al.*, 2023)

### Wellcome Sanger Institute – Legal and Governance

The materials that have contributed to this genome note have been supplied by a Darwin Tree of Life Partner. The submission of materials by a Darwin Tree of Life Partner is subject to the **‘Darwin Tree of Life Project Sampling Code of Practice’**, which can be found in full on the Darwin Tree of Life website here. By agreeing with and signing up to the Sampling Code of Practice, the Darwin Tree of Life Partner agrees they will meet the legal and ethical requirements and standards set out within this document in respect of all samples acquired for, and supplied to, the Darwin Tree of Life Project.

Further, the Wellcome Sanger Institute employs a process whereby due diligence is carried out proportionate to the nature of the materials themselves, and the circumstances under which they have been/are to be collected and provided for use. The purpose of this is to address and mitigate any potential legal and/or ethical implications of receipt and use of the materials as part of the research project, and to ensure that in doing so we align with best practice wherever possible. The overarching areas of consideration are:

•     Ethical review of provenance and sourcing of the material

•     Legality of collection, transfer and use (national and international) 

Each transfer of samples is further undertaken according to a Research Collaboration Agreement or Material Transfer Agreement entered into by the Darwin Tree of Life Partner, Genome Research Limited (operating as the Wellcome Sanger Institute), and in some circumstances other Darwin Tree of Life collaborators.

## Data Availability

European Nucleotide Archive: *Calliphora vicina* (urban bluebottle blowfly). Accession number PRJEB59784; https://identifiers.org/ena.embl/PRJEB59784 ( Wellcome Sanger Institute, 2023). The genome sequence is released openly for reuse. The *Calliphora vicina* genome sequencing initiative is part of the Darwin Tree of Life (DToL) project. All raw sequence data and the assembly have been deposited in INSDC databases. Raw data and assembly accession identifiers are reported in Table 1.

## Author information

Members of the Natural History Museum Genome Acquisition Lab are listed here: https://doi.org/10.5281/zenodo.7139035.

Members of the Darwin Tree of Life Barcoding collective are listed here: https://doi.org/10.5281/zenodo.4893703.

Members of the Wellcome Sanger Institute Tree of Life Management, Samples and Laboratory team are listed here: https://doi.org/10.5281/zenodo.10066175.

Members of Wellcome Sanger Institute Scientific Operations: Sequencing Operations are listed here: https://doi.org/10.5281/zenodo.10043364.

Members of the Wellcome Sanger Institute Tree of Life Core Informatics team are listed here: https://doi.org/10.5281/zenodo.10066637.

Members of the Tree of Life Core Informatics collective are listed here: https://doi.org/10.5281/zenodo.5013541.

Members of the Darwin Tree of Life Consortium are listed here: https://doi.org/10.5281/zenodo.4783558.
