# Supplementary material for: The genome sequence of a bluebottle fly, Calliphora vicina Robineau-Desvoidy, 1830
Source: Wellcome Open Res. 2025 Sep 3;9:335. Originally published 2024 Jun 24. [Version 2] doi: 10.12688/wellcomeopenres.22469.2 (PMC11519611; doi:10.12688/wellcomeopenres.22469.2)
Supplement: Peer review history

# Reviewer report: Reviewer response for version 1

**DOI:** 10.21956/wellcomeopenres.24757.r104421

**Published:** 2024-10-28

**Authors:** Paul V Hickner

**Affiliations:** 1 United States Department of Agriculture, Agricultural Research Service, TX, USA

I am a bit confused about the taxonomic authority, as the authors used Linnaeus, but others use Robineau-Desvoidy.

 If known, it would be appropriate to include the sex determination system. I believe they have an XX XY system (check Boyes and Shewell 1975), therefore the Y chromosome is not included in the assembly. Please acknowledge this. Otherwise, the quality of the genome assembly is very good. 

 A very minor issue, but "X sex chromosome" is not necessary, just X chromosome. 

Are sufficient details of methods and materials provided to allow replication by others?

Yes

Is the rationale for creating the dataset(s) clearly described?

Yes

Are the datasets clearly presented in a useable and accessible format?

Yes

Are the protocols appropriate and is the work technically sound?

Yes

Reviewer Expertise:

Calliphoridae genomics, behavior, and chemosensory gene family evolution.

I confirm that I have read this submission and believe that I have an appropriate level of expertise to confirm that it is of an acceptable scientific standard.

# Reviewer report: Reviewer response for version 1

**DOI:** 10.21956/wellcomeopenres.24757.r103333

**Published:** 2024-10-21

**Authors:** Denise Gemmellaro

**Affiliations:** 1 Department of Biology, Kean University, Union, New Jersey, USA

**BACKROUND**

 The morphological description of the species is ok, but considering the fly’s broad distribution it would be important to include information from other keys, perhaps even from different ecoregions, like Jones *et al* 2019. Also, I am not sure if I would use definitive color attributes (“blue”); since the fly is indeed metallic, it is possible to have different shades of perceived colors. Perhaps this could be solved by simply saying “blueish” instead of blue.

 A source is missing when talking about *C. vicina* being pollinators and attracted to a specific species of fungus.

 Considering that the genome presented in this indexed comes from a female specimen, it would be important to say how the authors determined the sex of the species (and relative source). Did they use the distance between the eyes or did they extract genitals?

 The indexed is highly informative in terms of content useful for taxonomy and phylogeny, but also because it presents a well-structured, easy to follow replicable protocols. I believe aside from minor adjustments suggested for the introduction, this paper is surely ready for indexing. Great work!

Are sufficient details of methods and materials provided to allow replication by others?

Yes

Is the rationale for creating the dataset(s) clearly described?

Yes

Are the datasets clearly presented in a useable and accessible format?

Yes

Are the protocols appropriate and is the work technically sound?

Yes

Reviewer Expertise:

Medico-legal forensic entomology, forensic science, molecular biology, ecology

I confirm that I have read this submission and believe that I have an appropriate level of expertise to confirm that it is of an acceptable scientific standard.
